# Disease-Related Changes in the Cerebrospinal Fluid Metabolome in Amyotrophic Lateral Sclerosis Detected by GC/TOFMS

**DOI:** 10.1371/journal.pone.0017947

**Published:** 2011-04-04

**Authors:** Anna Wuolikainen, Thomas Moritz, Stefan L. Marklund, Henrik Antti, Peter Munch Andersen

**Affiliations:** 1 Institute of Pharmacology and Clinical Neuroscience, Umeå University, Umeå, Sweden; 2 Department of Chemistry, Computational Life Science Cluster, Umeå University, Umeå, Sweden; 3 Department of Forest Genetics and Plant Physiology, Umeå Plant Science Centre, Swedish University of Agricultural Sciences, Umeå, Sweden; 4 Department of Medical Biosciences, Clinical Chemistry, Umeå University, Umeå, Sweden; Brigham and Women's Hospital, Harvard Medical School, United States of America

## Abstract

**Background/Aim:**

The changes in the cerebrospinal fluid (CSF) metabolome associated with the fatal neurodegenerative disease amyotrophic lateral sclerosis (ALS) are poorly understood and earlier smaller studies have shown conflicting results. The metabolomic methodology is suitable for screening large cohorts of samples. Global metabolomics can be used for detecting changes of metabolite concentrations in samples of fluids such as CSF.

**Methodology:**

Using gas chromatography coupled to mass spectrometry (GC/TOFMS) and multivariate statistical modeling, we simultaneously studied the metabolome signature of ∼120 small metabolites in the CSF of patients with ALS, stratified according to hereditary disposition and clinical subtypes of ALS in relation to controls.

**Principal Findings:**

The study is the first to report data validated over two sub-sets of ALS vs. control patients for a large set of metabolites analyzed by GC/TOFMS. We find that patients with sporadic amyotrophic lateral sclerosis (SALS) have a heterogeneous metabolite signature in the cerebrospinal fluid, in some patients being almost identical to controls. However, familial amyotrophic lateral sclerosis (FALS) without superoxide dismutase-1 gene (SOD1) mutation is less heterogeneous than SALS. The metabolome of the cerebrospinal fluid of 17 ALS patients with a SOD1 gene mutation was found to form a separate homogeneous group. Analysis of metabolites revealed that glutamate and glutamine were reduced, in particular in patients with a familial predisposition. There are significant differences in the metabolite profile and composition among patients with FALS, SALS and patients carrying a mutation in the SOD1 gene suggesting that the neurodegenerative process in different subtypes of ALS may be partially dissimilar.

**Conclusions/Significance:**

Patients with a genetic predisposition to amyotrophic lateral sclerosis have a more distinct and homogeneous signature than patients with a sporadic disease.

## Introduction

Amyotrophic lateral sclerosis (ALS) is an adult-onset heterogeneous syndrome with multiple clinical, genetic and histological subtypes with ill-defined borders [1], [2]. The clinical hallmarks of the syndrome are focal onset of symptoms and signs of degeneration of primarily upper and lower motor neurons, eventually leading to progressive generalized paresis of bulbar, limb, thoracic and abdominal skeletal muscles. Other brain functions, including oculomotor and autonomic functions, are relatively spared, though these may be involved in some patients. Cognitive dysfunction occurs in 25–50%, and 5–15% of patients develop dementia that is usually of frontotemporal type; semantic dementia or progressive non-fluent aphasia. In epidemiological studies in different populations, familial ALS (FALS) is reported by 1–18% of patients, and presently mutations in ten genes have been found to cause ALS. The most commonly mutated gene in FALS is the gene encoding the oxygen-radical scavenging enzyme superoxide dismutase-1 (SOD1), mutations in which have been found in 12–23% of patients with a diagnosis of FALS and in 1–7% of patients carrying a diagnosis of sporadic ALS (SALS)[3]. The other genes found mutated in ALS have been found in 2% or less of cases and include genes involved in axonal transportation, angiogenesis and RNA processing without a common denominator except for the ALS phenotype. Genomic studies suggest the existence of at least eleven additional loci for FALS, but the genetic defects remain to be identified [3].

Post-mortem histological hallmarks of ALS are reduction in number of spinal anterior horn motor neurons and the presence of Bunina bodies and ubiquinated cytoplasmic inclusions in the remaining spinal motor neurons. These inclusions may be immunoreactive to antibodies against the TAR DNA binding protein (TDP-43) and p62. Interestingly, immunoreactivity to TDP-43 appears to be rare or absent in patients with SOD1 gene mutations suggesting that these may follow a different neurodegenerative pathway [4], [5].

ALS is invariably fatal, the median survival time is 2.5 years before the patient succumbs, usually to paralysis of the respiratory muscles. Epidemiological and neurophysiological studies and studies in transgenic rodent models with mutations in the SOD1 gene suggest that the actual pathogenic process begins much earlier and not only involves neurons but also T-cell and glial cell populations as well as endothelial cells involved in maintaining the blood-brain barrier [6].

A number of studies of plasma, serum and/or cerebrospinal fluid (CSF) have failed to identify the key components in the neurodegenerative process in ALS. Series of transcriptomic and proteomic studies have given diverging and frequently inconclusive results with few validated early findings. Reduced levels of cystatin C and increased levels of neurofilaments have been proposed as CSF biomakers for ALS [7], [8]. A recent proteomic study found a relationship between the levels of Galectin-3 to be indicative of the onset of ALS symptoms in mice and the result was found transferrable to ALS [9]. Metabolomics should be seen as a complementary technique to genomics, transcriptomics and proteomics. In 2005, the first metabolomic study of ALS reported reduced levels of a broad variety of metabolites in blood plasma although the results were inconclusive [10]. Recently, Blasco *et al.* [11] reported a proton nuclear magnetic resonance (^1^H-NMR) based metabolomic study of CSF from 50 ALS patients and 44 controls (non-neurological diseases). Acetone, pyruvate and ascorbate were found significantly increased while acetate was found decreased in ALS compared to controls, respectively. In this interesting study, only a small part of the metabolome was studied. The authors concluded that perturbations in energy metabolism and ketone metabolism were associated with ALS. However, other earlier studies of ascorbate metabolism have showed contradictory results [12], [13]. We here report a carefully designed study of the human CSF metabolome in a larger group of patients with different clinical and genetic subtypes of ALS. This study reports data validated over two sub-sets of ALS vs. control patients for a large set of metabolites analyzed by GC/TOFMS.

## Methods

### Sample collection, handling and storage

The study was performed in accordance with the Declaration of Helsinki (WMA, 1964) and approved by the medical ethical research board at the University of Umeå, Sweden. With written informed consent CSF was collected from ALS patients divided into two groups (set I, set II), each group consisting of 39 ALS patients: Set I (19 males, 20 females; 30 SALS and 9 FALS; 8 with bulbar onset, 31 with spinal onset; 6 with SOD1 gene mutations) and Set II (20 males, 19 females; 25 SALS and 14 FALS; 9 with bulbar onset, 30 with spinal onset; 12 with SOD1 gene mutations). The ALS patients were diagnosed by the same neurologist according to standard criteria [3]. Cases were classified as FALS when two, or several members in the same family had been diagnosed with ALS. The remaining cases were classified as SALS. The selection process is summarized in Figure S1 and the characteristics of each patient and their respective controls are summarized in Table S1. For each patient, one or more control individual(s) were carefully selected matched for age, gender and the time the CSF sample had been in freezer storage. In set I, some of the ALS patients were assigned to match more than one control to account for the possibility that the matched control may have a disease that could show differences in the metabolite pattern towards ALS. Multiple ALS subjects were also matched to the same control to account for the inverse, i.e that different ALS subtypes may show a different metabolic response compared to the same control. In set II, only one control was matched to each ALS patient. The control patients either suffered from a neurological condition or were healthy subjects. Details for each ALS-patient and the corresponding control are summarized in Table S1. The CSF was tapped as part of the initial clinical investigation before treatment with riluzole was initiated. In a few patients, the spinal tap was performed as part of a secondary investigation and these patients were usually already medicating with riluzole (Table S1). In all patients, a spinal tap was performed at the L:4-L:5 or L:5-LS1 levels with the individual lying in a horizontal resting fetal position (right side down). The spinal taps were performed non-traumatically without visible or laboratory evidence (enumeration in a Bürker Champer) for hemorrhage using a 20 G Spinocan® cannula (patients in which even the smallest hemorrhage in the CSF was detected were excluded from the study) as described [14]. The CSF used in the present study was among the first 4 ml collected. Upon collection, the CSF was immediately frozen in 1 ml polypropylene tubes to −80°C as described [14]. None of the individuals were fasting at the time of the tapping. The ALS patients were stratified according to clinical subtype of ALS (gender, bulbar or spinal onset, familial or sporadic disease) and results of genetic analysis (SOD1 mutation/non-SOD1 mutation).

### Genetic analysis

All patients were screened for DNA-mutations in the following genes: SOD1, vesicle associated membrane protein B, TDP-43, fused in sarcoma (FUS), progranulin, chromatin modifying protein 2B, optineurin, ataxin 2 and angiogenin using standard procedures as described [15], [16], [17], [18], [19], [20], [21], [22], [23]. Only patients with a SOD1 gene mutation or patients without a mutation in any of the analyzed genes were included in this study.

### Metabolomic analysis

CSF from the two groups (set I and set II) of ALS patients (39+39) and their matching controls were analyzed using gas chromatography - time of flight mass spectrometry (GC/TOFMS) with 6 months in between. Division into the two sample sets (I and II) was done to validate disease related findings in two separate cohorts over time. Biological (samples from the same subject extracted, derivatized and analyzed separatelt) and analytical (the same sample being injected on the GC multiple times) replicates were made in both sets to assess the stability of the method for both control and ALS subjects. In order to obtain a reasonable number of samples in each GC/MS run only a selection of the samples were replicated. Samples from patients and controls were analyzed in a randomized order in set I, and in a pair-wise randomized order in set II where permutation was made to decide which sample was first analyzed for each pair. The combination of the two methods for validation was employed to minimize the risk to get results based on spurious correlations from the data collection.

Extraction of CSF, derivatization of extracted metabolites, GC/TOFMS analysis, data pre-processing and metabolite identification were carried as outlined in Figure S2 and described in Methods S1 and [14]. The study was performed in accordance with the standards proposed by the Chemical Analysis Working Group and Metabolomics Standards Initiative for chemical analysis and practices related to analysis of the metabolome [24].

### Data analysis

Multivariate statistical modeling was performed by means of orthogonal partial least squares discriminant analysis (OPLS-DA) [25]. OPLS was developed to deal with complex data consisting of systematic variation from multiple origins (biological, analytical, etc.), such as metabolomic data, by splitting information in one part correlated to a certain response (for example disease status), and one part uncorrelated (i.e. orthogonal) to the response. This provides OPLS with the advantage, compared to other generalized inverse regression models, that it facilitates model interpretation and visualizations of both types of variations [26]. A special case of OPLS is when the response is constructed as dummy variables holding information about the sample class called discriminant analysis (DA). Variables (metabolites) are extracted that discriminates between the sample classes (the method is hence called OPLS-DA). For details regarding OPLS-DA please see Methods S1 and Figures S3 and S4 [25].

Validation of models was done using full cross validation (CV), CV-analysis of variance [27] and in some cases prediction of model independent samples. In detail, models were calculated in set I and set II for the differences between *i)* ALS and control, *ii)* FALS and control, *iii)* SALS and control, for set I and set II. For specific sub-group comparisons of all ALS cases (FALS/SALS, SOD1 mutation/non-SOD1 mutation) a third set (set III) was constructed based on set I and set II. The same data pretreatment was used for set I as for set II and for each ALS subject the matched control was subtracted. For set III models were calculated for the differences between *i)* FALS and SALS and *ii)* ALS cases with and without SOD1 mutations. For models revealing significant disease-related separations important metabolites were highlighted and subjected to further validation. All variables were used for modeling of training set and predictions (centered and scaled to unit variance). All multivariate statistical modeling was carried out in SIMCA P+12.0 (Umetrics, Umeå, Sweden). Model plots were created using Evince 2.3.1 (UmBio AB, Umeå, Sweden).

The total sum of variables (artifacts, exogenous compounds and internal standards excluded) was calculated for each subject. T-test was used to calculate p-values for the differences between *i)* ALS and control, *ii)* FALS and matched controls, *iii)* SALS and matched controls, *iv)* FALS and SALS and *v)* matched controls for FALS and matched controls for SALS of the total sum of variables. For SOD1 mutations no test were performed due to the small number of individuals.

## Results

### ALS versus controls

**Figure 1:**
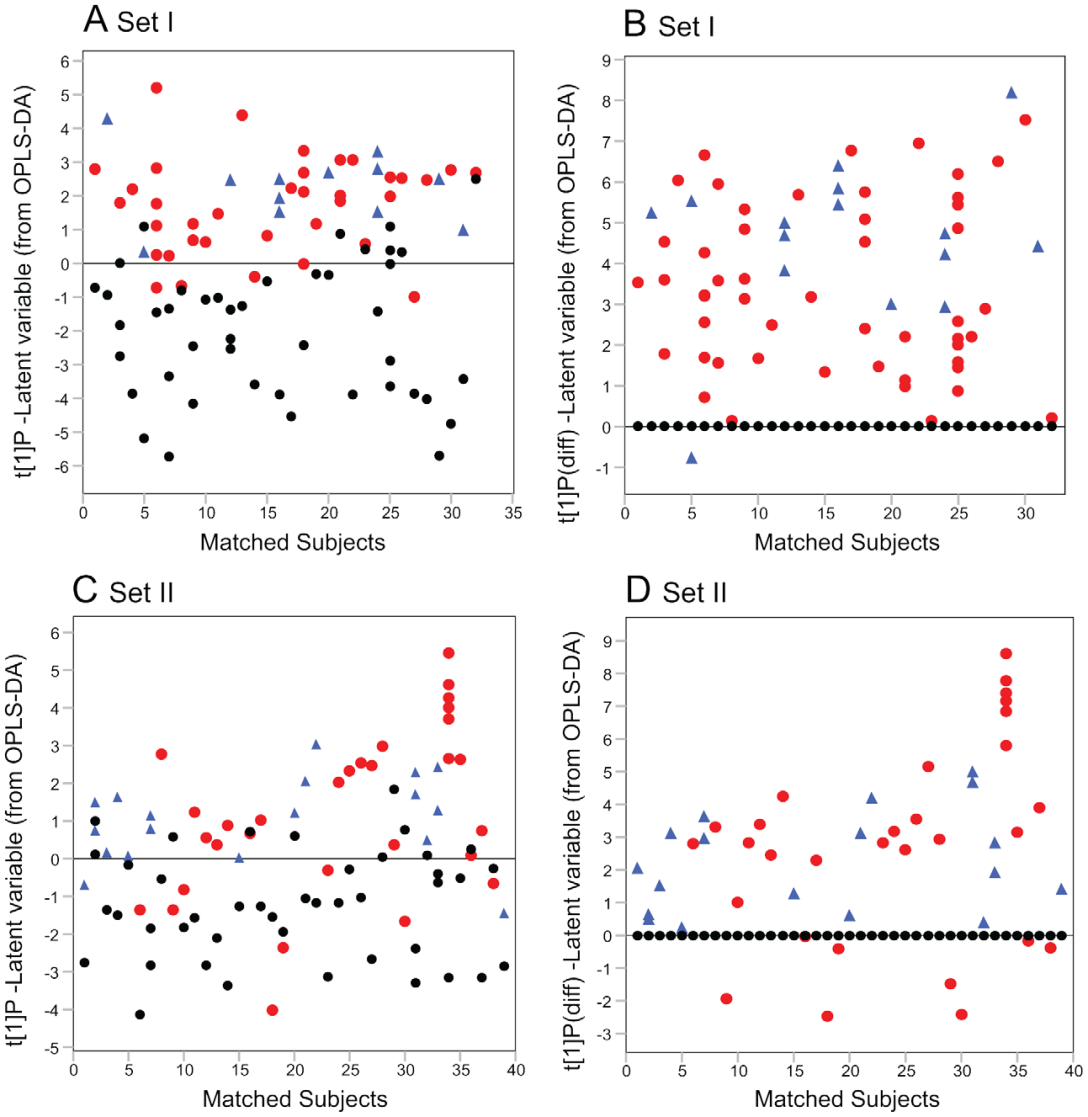
OPLS-DA model results for all ALS versus controls. (A) OPLS-DA model score vector t[1]P vs. matched sample pairs showing separation between controls and ALS (SALS and FALS) in set I. (B) Showing the separation between controls and ALS samples (SALS and FALS) after subtraction of control score values from matched ALS case score value in set I. (C) OPLS-DA model score vector t[1]P vs. matched sample pairs showing separation between controls and ALS samples (SALS and FALS) in set II. (D) The separation between control and ALS samples (SALS and FALS) after subtraction of control score values from matched ALS score values in set II. Red circles, SALS; blue triangles, FALS; black circles, control.

OPLS discriminant analysis (OPLS-DA) was used to screen the data for alterations in metabolites related to disease classification (control versus ALS). Modeling of the two datasets I and II separately, revealed separation/overlap between subjects diagnosed with ALS compared to their matched controls. The separation was more distinct in set I as compared to set II (Fig. 1) and it was found that the separation was caused by a few samples (mainly SALS cases) not following the common disease pattern (Fig. 1 a and c). The common disease pattern was further enhanced by subtracting the model score values for the matched control subject from each corresponding ALS case to give all control samples a common starting value of zero. ALS samples systematically move in a common ALS direction in the “metabolic space”. In set I, one FALS subject was misclassified when compared to a control subject diagnosed with chronic fatigue syndrome. The same FALS was correctly classified compared to a healthy control (1 misclassified/39 in set I). In set II seven SALS cases were misclassified (7 misclassified/39 in set II). The pattern was hence generally more consistent for samples classified as FALS as compared to cases classified as SALS (Fig. 1 b and d). FALS was found to be a more homogenous group in the obtained model scores (set I: standard deviation (SD) = ±1.21; set II: SD = ±1.14) compared to SALS (set I: SD = ±1.38; set II: SD = ±2.22).

### Subdivision into familial and sporadic ALS cases

**Figure 2:**
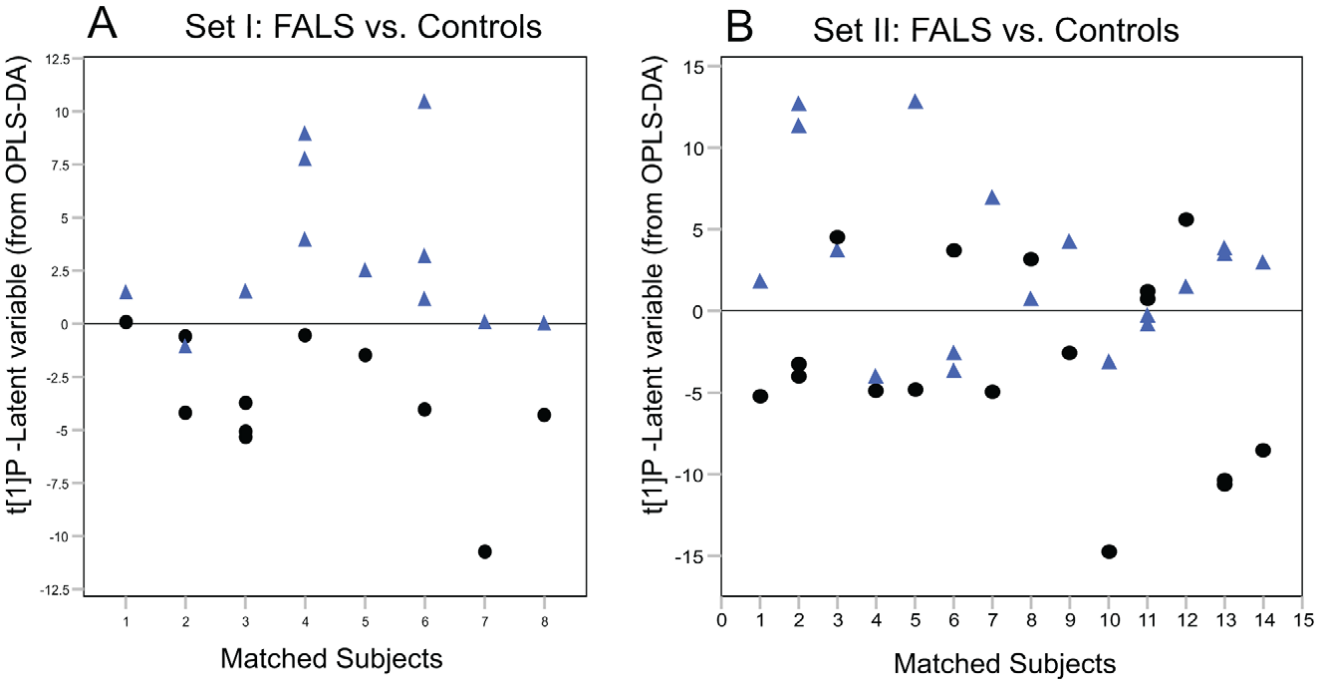
OPLS-DA model of FALS versus controls. OPLS-DA model score vector t[1]P vs. matched sample pairs showing separation between control (black circles) and FALS samples (blue triangles) for set I (A) and for set II (B).

To address more specific changes of SALS and FALS respectively in the CSF metabolome, the individual groups where modeled together with the corresponding matched controls. OPLS-DA models between FALS and the matched controls revealed a significant separation between the FALS cases from the matched control in both set I and set II (Fig. 2). However, for the SALS cases significant separation from controls was only found in set I. This was in accordance to what was seen when modeling ALS against controls.

**Figure 3:**
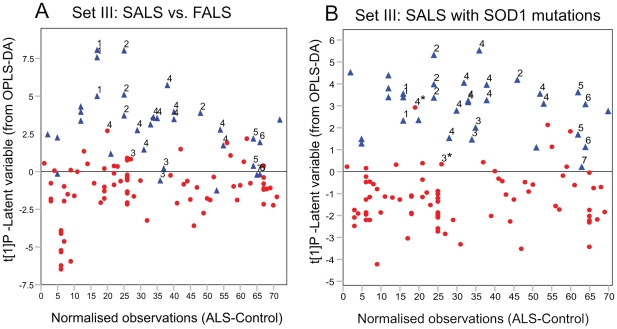
OPLS-DA model of SALS versus FALS. (A) OPLS-DA model score vector t[1]P for the model between SALS samples and FALS samples showing a clear difference between the sample groups. Two patients later found to carry SOD1 mutations (D90Amc and D90Amm) were initially diagnosed as apparently SALS. (B) OPLS-DA model score vector t[1]P for the model between SALS samples and FALS samples when excluding the two SALS patients carrying SOD1 mutations (D90Amc and D90Amm). Predictions of the two excluded samples (marked with stars) placed them into the FALS group. Blue triangles, FALS; red circles, SALS.

The FALS and SALS cases from both set I and II were then included in the same model following subtraction of the matched control values from the corresponding ALS subject (named set III). Comparison of FALS and SALS cases revealed a significant difference between the two ALS subtypes (Fig. 3 a). In addition, when excluding two subjects classified as SALS but carrying a SOD1 mutation an even stronger model for the separation was obtained. Prediction of the two excluded SALS cases (SOD1 positive) into the model placed them closer to the group of FALS cases as compared to SALS (Fig. 3 b).

### Subdivision into ALS cases with and without SOD1 mutations

**Figure 4:**
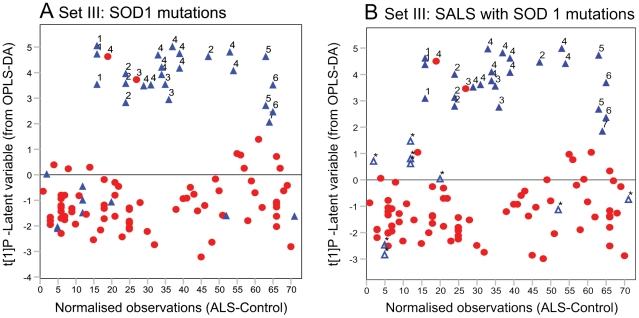
OPLS-DA results for patients with SOD1 gene mutations. (A) OPLS-DA score vector t[1]P showing the difference between ALS samples with (blue triangles) and without (red circles) mutation in the SOD1 gene. (B) Prediction of six FALS cases (some replicated) without SOD1 mutation (open blue triangles marked with stars) into an OPLS-DA model between samples with mutation in the SOD1 gene and SALS samples without mutation in the SOD1 gene places the four FALS cases without mutation in the non-SOD1 group and two on the borderline between the groups (samples predicted to have a positive value along tPS [1]).

Seventeen of the samples were from patients with one of 6 different SOD1 gene mutations (please see Table S1 for details). Comparison of the metabolome of these ALS patients to the SALS and FALS patients without SOD1 mutations, revealed a significant separation between the groups (Fig. 4 a). Furthermore, a significant separation was found when comparing carriers of a mutation in the SOD1 gene (FALS and SALS) to SALS cases negative for SOD1 mutations (hence excluding all SOD1 negative FALS cases). Prediction of SOD1 mutation negative FALS cases into the model placed four out of six samples in the group of SALS cases negative for SOD1 mutations while two samples ended up on the border between the groups (Fig. 4 b).

For a summary of the statistics of all calculated OPLS-DA models see Table 1.

**Table 1:** OPLS-DA model diagnostics.

Model number	Model between	Dataset	Number of model components	R2X (t1P)	R2X	R2Y	Q2	CV-ANOVA
1	ALS-Controls	Set I	1+1+0	0,050	0,162	0,567	0,311	8,52*10^-7^
2	ALS-Controls	Set II	1+1+0	0,044	0,306	0,340	-0,143	n.s.
3	FALS-Controls	Set I	1+0+0	0,201	0,201	0,529	0,265	4,77*10^-2^
4	FALS-Controls	Set II	1+2+0	0,061	0,453	0,896	0,400	4,73*10^-2^
5	SALS-Controls	Set I	1+1+0	0,055	0,175	0,604	0,236	2,15*10^-3^
6	SALS-Controls	Set II	1+0+0	0,209	0,209	0,238	-0,136	n.s.
7	FALS-SALS	Set I	1+0+0	0,148	0,148	0,396	0,263	7,70*10^-4^
8	FALS-SALS	Set II	1+1+0	0,146	0,345	0,730	0,411	1,13*10^-4^
9	FALS-SALS	Set III	1+1+0	0,064	0,220	0,475	0,219	2,65*10^-5^
10	FALS-SALS (prediction of mutation SALS)	Set III	1+3+0	0,036	0,332	0,732	0,316	4,91*10^-6^
11	SOD1-nonSOD1	Set III	1+4+0	0,037	0,358	0,860	0,428	9,35*10^-9^
12	SOD1-nonSOD1 (prediction of FALS)	Set III	1+1+0	0,070	0,223	0,542	0,325	8,76*10^-8^

R2X (t1P): How much of the variation in X is directly related to Y; predictive variation in X. R2X: How much of the variation in X is explained by the model. R2Y: How much of the variation in Y is explained by the model. Q2: How much of the variation in Y can be predicted by the model. CV-ANOVA: P-value for the separation between sample groups based on cross validated model scores.

### Metabolite detection and identification

In total 120 peaks (e.g. potential metabolites) were detected with GC/TOFMS of which forty metabolites were identified. Eighty peaks were found to be potential metabolites but could not be identified with high enough certainty using chemical reference libraries. Around thirty of the eighty unidentified compounds could still be assigned to compound class. The overall alterations including metabolites highlighted as contributing to the significant separations in the models reported above are summarized in Table S2. The total sum of variables was found to be significantly different between the group of FALS patients and the group of matched controls (p <0.05 for both set I and set II) as well as between FALS and SALS (p <0.05 for both set I and set II).

## Discussion

The metabolome consists of highly covarying metabolites [28]. This is to date the largest study to cover a larger proportion of the human CSF metabolome in patients with a neurodegenerative disease compared to controls. Earlier metabolomic studies in ALS have focused on a few metabolites like glutamic acid, aspartic acid, homocysteine [29], creatinine, lactic acid/pyruvic acid, lipid metabolism and not on metabolomic profiling as in the present study. In the earlier reports, no consistent abnormality has been found validated over several studies. Using ^1^H-NMR to study 17 metabolites in the CSF from 50 ALS patients, Blasco *et al.* recently found significantly lower acetate concentrations, a trend for an increase in acetone, and significant increases in the concentrations of both pyruvate and ascorbate in ALS compared to 44 controls [22]. Although ^1^H-NMR spectroscopy is a robust method for analysis of many metabolites, GC/TOFMS has the advantage to discover changes in metabolites present at lower concentrations due to the increased sensitivity (for instance, to analyze metabolites such as glutamic acid) hence NMR and mass spectrometry should be regarded as complementary techniques. Glutamic acid was in the present study found to be reduced in CSF from the ALS patients. In earlier studies performed over the past twenty years and using different methodologies and usually only a small number of patient samples, glutamic acid has been reported as being elevated, normal or reduced in the CSF of ALS patients [30], [31], [32]. The studies reporting an increase in CSF glutamic acid content has by some been taken as support for the theory of glutamic acid exitotoxicity as a cause of ALS [30]. We have recently reported that glutamic acid in CSF is highly susceptible to sampling variables and in particular to storage temperature (−20°C versus −80°C) [14]. Higher values of glutamic acid are found in CSF samples stored even for a short time at room temperature or −20°C. In our experience, rigorous quick handling of freshly drawn CSF, low storage temperature and the careful matching of stored ALS and control samples are essential for reliable assessment of glutamic acid and other metabolites in the CSF.

In contrast to the interesting study by Blasco *et al.* [11], we find that the content of ascorbic acid to be non-significantly different between ALS and controls in both set I and set II (non-significant increased in set I, non-significant decreased in set II).

Creatinine was reduced in the ALS patients. The sources of the compound, creatine and creatine phosphate, are important for the energy metabolism and are present at high levels in the CNS. The major formation of creatinine in the body, however, takes place in the skeletal muscles. CSF creatinine levels reflect both local production of the compound and plasma creatinine levels [33]. This suggests that amyotrophy is the most likely explanation to the lower CSF creatinine levels in ALS.

The patients with 6 SOD1 gene mutations with different clinical and genetic subtypes (dominant versus recessive inheritance) of ALS and very variable survival times had a rather uniform metabolomic signature suggesting a common neurodegenerative pathway for patients with this type of ALS, but no single abnormal metabolite could be identified. The 6 FALS patients without a SOD1 gene mutation were less homogeneous than the mutant SOD1 group, and notably did not overlap with this group, possibly because of alternative degenerative pathways. Unfortunately, no CSF samples fulfilling the stringent sampling criteria used in this study were available from patients with mutations in other identified ALS genes. The patients with a SALS diagnosis without a SOD1 gene mutation appeared as a more heterogeneous group possibly because of multiple disease pathways. Some overlap between SALS with the control group was found. The 8 SALS cases that were most "control-like” did not share any common other features, including co-existing or previous other diseases or medication. Three of these SALS cases underwent post-mortem examination confirming the ALS diagnosis.

The results indicate larger heterogeneity in metabolite patterns among SALS cases compared to FALS, which were found to be more defined as a group. One may speculate whether the results of the study point towards either ALS as a group of diseases or one disease showing diverse metabolic fluctuations due to various combinations of symptoms related to the same disease or alterations due to different rates of progression of the disease. The variability may be one of the reasons for the relatively poor model diagnostics when comparing ALS to controls and SALS to controls. Other reasons may be the large diversity within the control group consisting of patients of various neurological diseases and errors of diagnosis (false positives or false negatives). Another possibility is that the pathology in ALS is to only a limited extent reflected in the sub-fraction of the CSF-metabolome captured in the study.

However, some general systematic patterns possibly related to disease were found. An overall trend was decreased levels of many CSF metabolites in ALS. This is in accordance with the interesting recent study of the plasma metabolome in ALS versus controls [10]. Further analyses of the still non-covered part of the metabolome and prospective studies are needed to draw general mechanistic conclusions regarding disease related changes.

### Limitations of the study

Riluzole is the only drug that has been shown to slow the course of ALS in class I studies, by some attributed to its anti-glutamatergic properties but the mechanism is unknown [34]. While most of the patients included in this study were without any medication at the time of CSF collection, a few had been on riluzole for weeks or months when the spinal tap was performed as part of a second opinion evaluation (Table S1). Riluzole was not detected in the CSF by GC/TOFMS, although there is a possibility that metabolites of the drug may be present as non-identified metabolites, as could effects of the drug on the endogenous metabolome be present as a factor in the treated patients. However, the design of the study including both riluzole-treated and untreated ALS cases diminishes the risk that the detected disease related model separations should be caused by direct or indirect riluzole-induced metabolic alterations.

The present metabolomics study is based on GC/TOFMS analysis. This type of analysis has its limitation as it can only cover approximately 10–15% of the metabolome, and then mainly primary metabolites such as amino acids, fatty acids, other organic acids, mono- and disaccharides. Today no single analytical platform can analyze the whole metabolome. Instead a combination of GC/MS, LC/MS and NMR are required to cover an extended proportion of the entire metabolome. Such a global metabolomics study will be of the greatest interest but will demand extensive laboratory resources and large volumes of CSF. It must also be emphasized that many metabolites are still unidentified, and therefore identification of metabolites in biological fluids such as CSF will be of major importance for the future.

One advantage of using GC/TOFMS is that many compounds can comparatively easily be identified, yet still only 30% of the detected metabolites could be identified in this study (Table S2). Obviously we are only at the beginning of tapping the wealth of information contained in the metabolome in diseases.

## Supporting Information

Figure S1 **Algorithm for sample selection**. Samples from ALS subjects were ordered according to age and storage time in -80°C freezer for males and females separately and two subsets were selected and merged. Controls with various neurological conditions and healthy subjects were matched according to sex, age and storage time.
(TIFF)

Figure S2 **Derivatization algorithm.** The selected CSF samples were extracted and derivatized according to the scheme above prior to GC-TOFMS analysis.
(TIFF)

Figure S3 **OPLS score vector t1[p] vs. matched sample pairs**. The figure present the separation between controls (open squares) and matched ALS subjects (black squares) for group A (left) and group B (right) based on an OPLS model against GC-TOFMS run order showing nonsystematic patterns related to disease.
(TIFF)

Figure S4 **Algorithm for Data processing.** Settings of HMCR were optimized according to a full factorial design. Max peak shift setting was kept constant at 45 scans while, filter length, noise limit and retention time precision were adjusted according to the paradigm in the figure. Nine datasets were evaluated for number of resolved components, number of bad spectra (estimated by median intensity of *m/z* values ≠ 0), number of split peaks of internal standards, match according to library search of internal standards and a selection of endogenous metabolites.
(TIFF)

Table S1 **Characteristics of Patients and Controls in Dataset I and Dataset II** Abbreviations used: FTD, Frontotemporal dementia; NPH, Normal pressure hydrocephalus; PBP, Progressive Bulbar Palsy; SBMA, Spino-bulbar muscular atrophy; FALS, familial ALS; SALS, sporadic ALS; wt, wild-type SOD1 genotype; D90A/D90A, homozygosity for the D90A SOD1 allele (mm); ALSFRS, ALS Functioning Rating Scale (40 is the score for a healthy individual).
(XLS)

Table S2 **Identified Metabolites** Identified metabolites from set I and II contributing to differences in one or several comparisons. Contribution in models of ALS-Controls, FALS-Controls, FALS-SALS and mSOD1-wtSOD1 are shown. Abbreviations: Loadings (p1)*; Contribution (w*1)**; Exogenous***; −/+  =  decreased or increased in ALS, FALS or patients with a SOD1 gene mutations; 0  =  no change found; nf  =  not found by analysis; blank  =  not found after cutoff 2/3.
(XLS)

Methods S1 (DOC)
