# Supplementary figures and images for: Disease-Related Changes in the Cerebrospinal Fluid Metabolome in Amyotrophic Lateral Sclerosis Detected by GC/TOFMS
Source: PLoS One. 2011 Apr 4;6(4):e17947. doi: 10.1371/journal.pone.0017947 (PMC3070699; doi:10.1371/journal.pone.0017947)

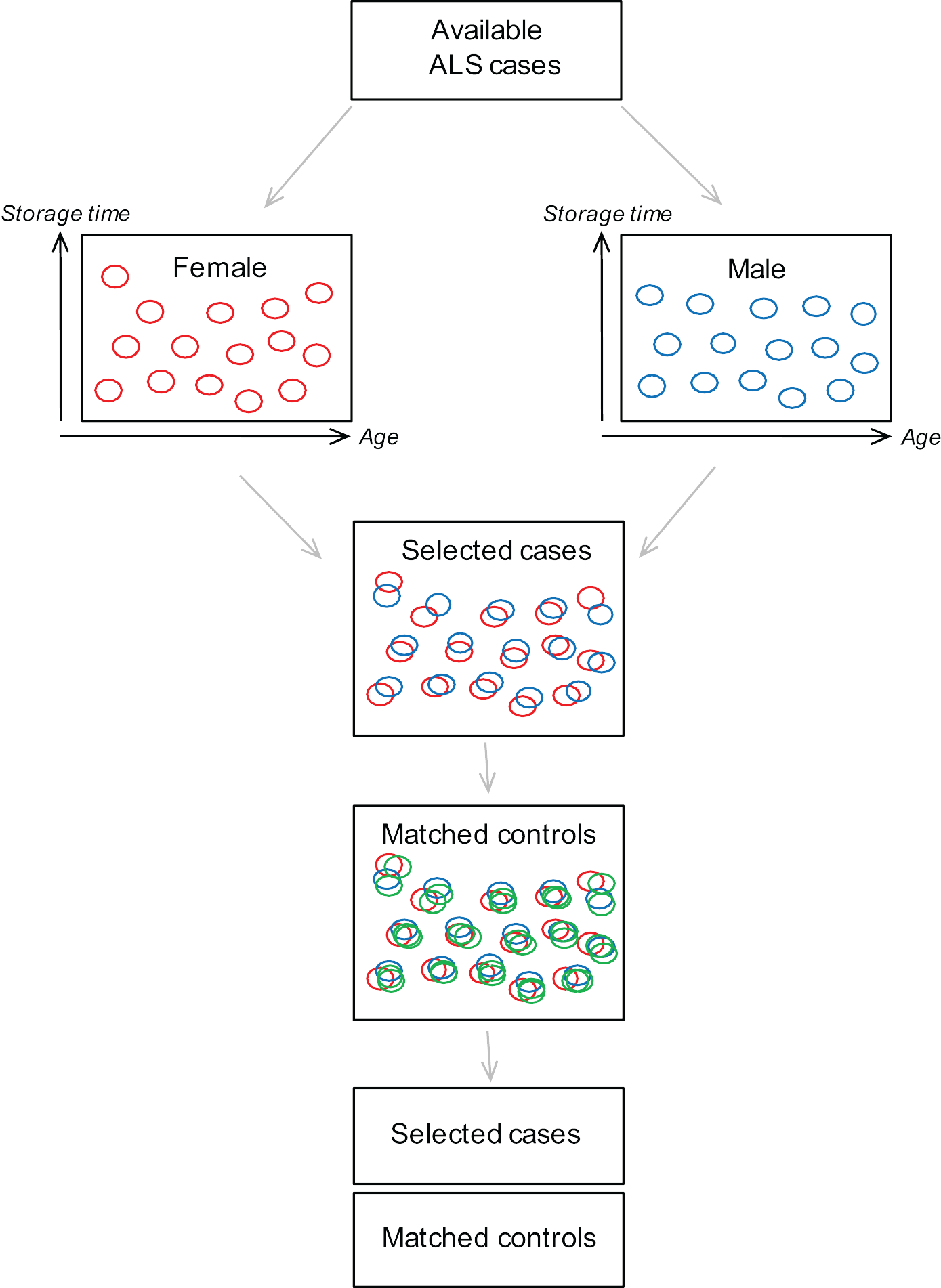

Supplement: Figure S1 — Algorithm for sample selection. Samples from ALS subjects were ordered according to age and storage time in -80°C freezer for males and females separately and two subsets were selected and merged. Controls with various neurological conditions and healthy subjects were matched according to sex, age and storage time. (TIFF) [file pone.0017947.s001.tiff]

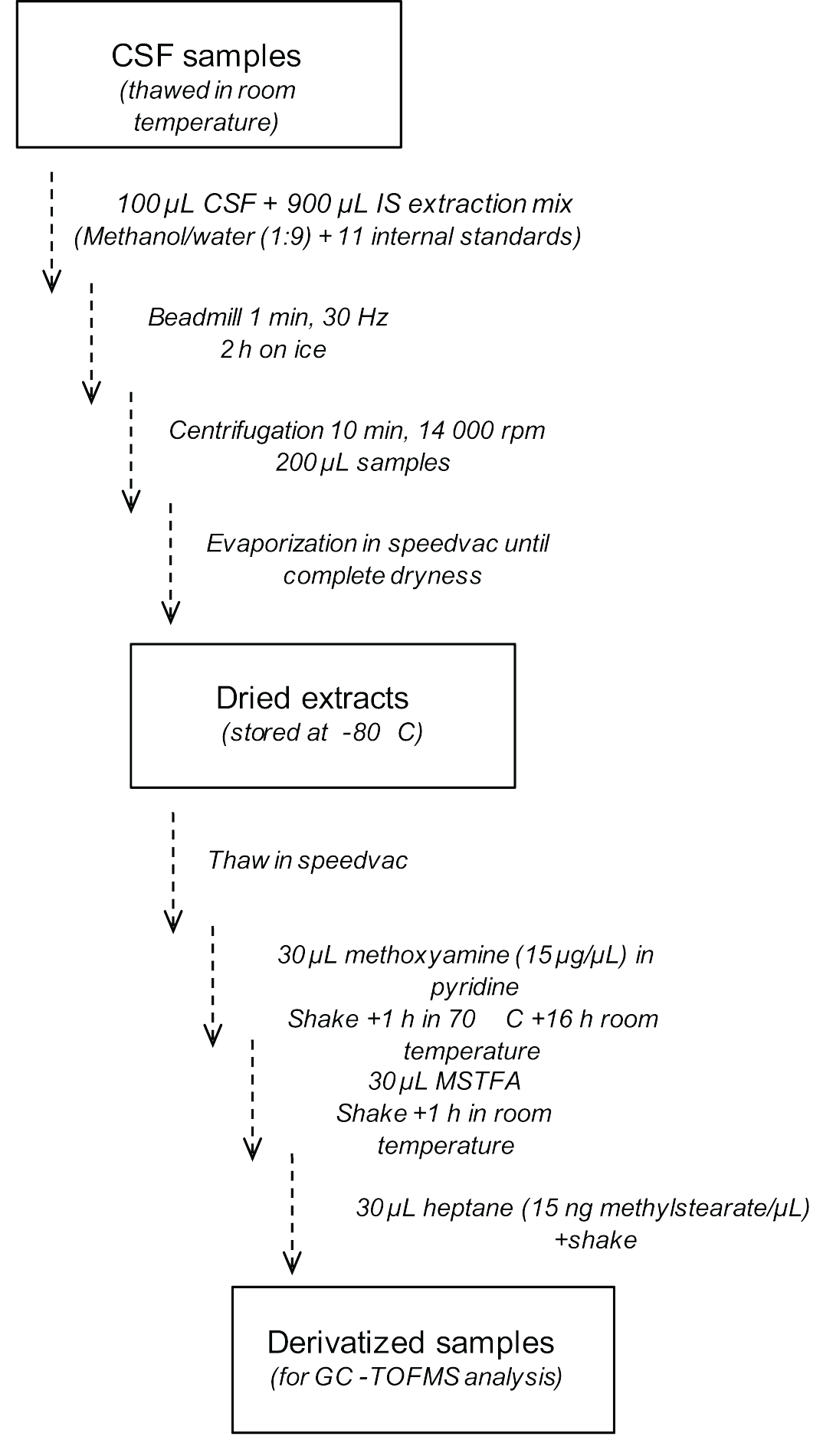

Supplement: Figure S2 — Derivatization algorithm. The selected CSF samples were extracted and derivatized according to the scheme above prior to GC-TOFMS analysis. (TIFF) [file pone.0017947.s002.tiff]

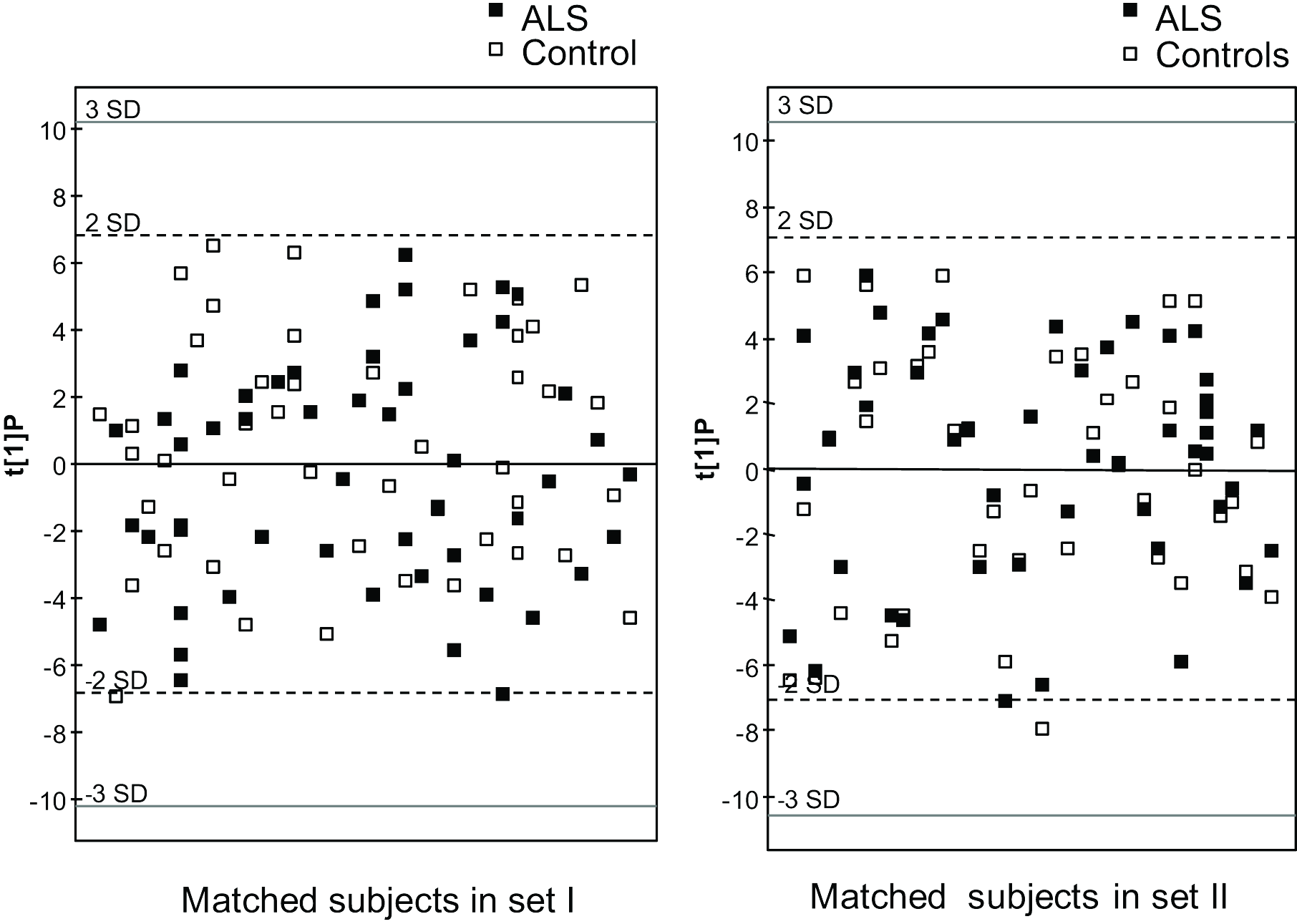

Supplement: Figure S3 — OPLS score vector t1[p] vs. matched sample pairs. The figure present the separation between controls (open squares) and matched ALS subjects (black squares) for group A (left) and group B (right) based on an OPLS model against GC-TOFMS run order showing nonsystematic patterns related to disease. (TIFF) [file pone.0017947.s003.tiff]

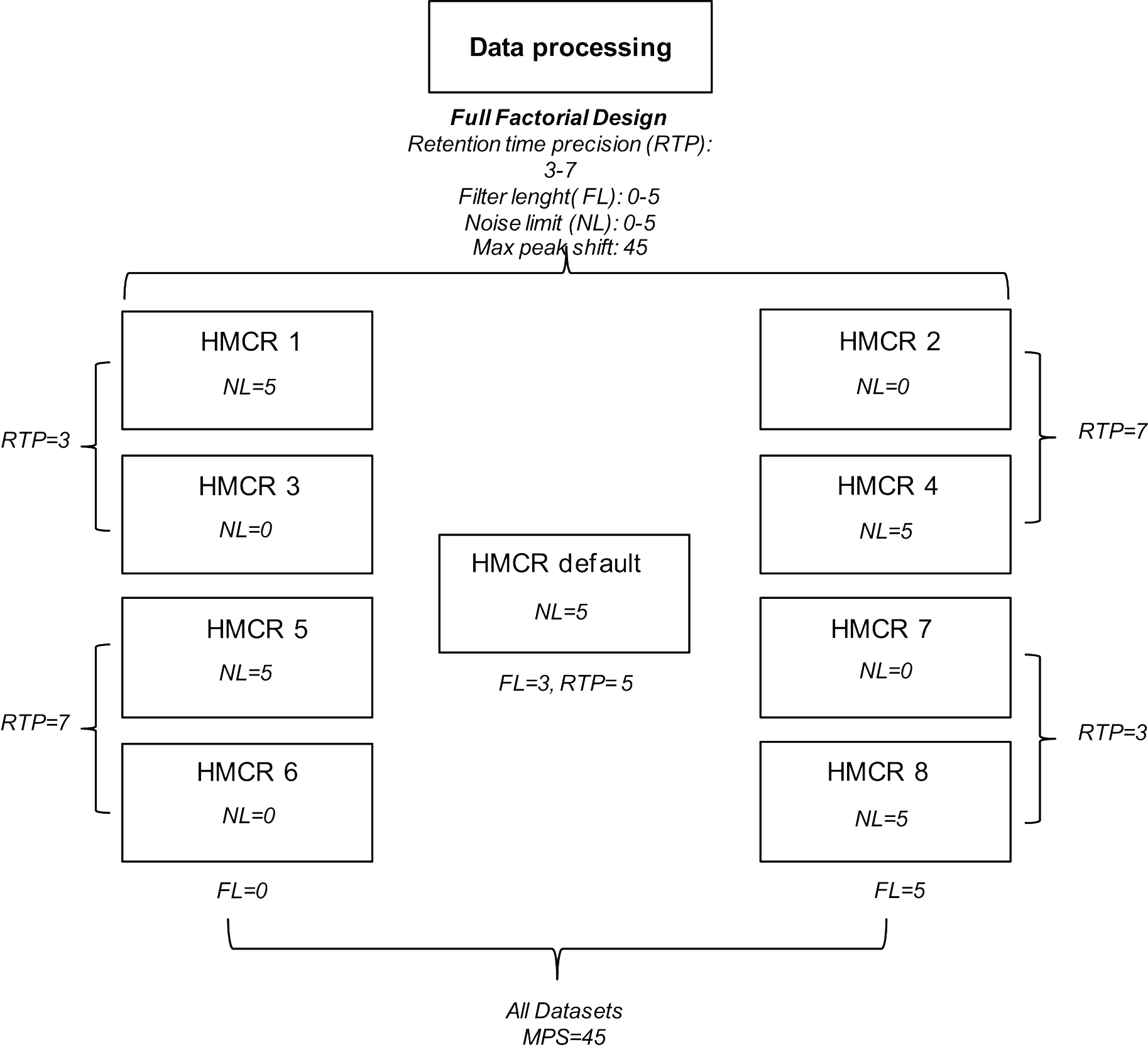

Supplement: Figure S4 — Algorithm for Data processing. Settings of HMCR were optimized according to a full factorial design. Max peak shift setting was kept constant at 45 scans while, filter length, noise limit and retention time precision were adjusted according to the paradigm in the figure. Nine datasets were evaluated for number of resolved components, number of bad spectra (estimated by median intensity of m/z values ≠ 0), number of split peaks of internal standards, match according to library search of internal standards and a selection of endogenous metabolites. (TIFF) [file pone.0017947.s004.tiff]
