# Supplementary material for: Disease-Related Changes in the Cerebrospinal Fluid Metabolome in Amyotrophic Lateral Sclerosis Detected by GC/TOFMS
Source: PLoS One. 2011 Apr 4;6(4):e17947. doi: 10.1371/journal.pone.0017947 (PMC3070699; doi:10.1371/journal.pone.0017947)
Supplement: Methods S1 — (DOC) [file pone.0017947.s007.doc]

SUPPORTING DOCUMENT: METHODOLOGY

**Selection of samples**

The selection process is outlined in Figure S1.

**Extraction of small molecules from CSF**

To prepare the extraction mix methanol/water 8:1 was supplied with stable isotope-labelled internal standards (IS). *Water soluble IS:* [^2^H_4_] butanediamine ·2HCl (500 ng/μL), [^13^C_4_] disodium-α-ketoglutarate (500 ng/μL), [^13^C_6_] glucose (500 ng/μL), [^13^C_5_,^15^N] glutamic acid (500 ng/μL), [^13^C_5_] proline (500 ng/μL), [^2^H_4_] succinic acid (500 ng/μL), [^13^C_12_] sucrose (500 ng/μL). *Methanol soluble IS:* [^2^H_7_] cholesterol (500 ng/μL), [1,2,3-^13^C_3_] myristic acid (500 ng/μL), [^13^C_4_] palmitic acid (500 ng/μL) and [^2^H_6_] salicylic acid (500 ng/μL). Final concentration of each IS in the extraction solution was 7 ng/μL.

**Derivatization of dried extracts**

*Step 1:* *O*-methylhydroxylamine hydrochloride 98% (Aldrich) (30 μL, 15 μg/μl) in pyridine was added to the samples, shaken with a MM301 beadmill (Retsch® GmbH & Co. KG) for 15min, heated to 70 °C for 1 h and left for 16 h in room temperature.
*Step 2:* N-Methyl-*N*-trimethylsilyltrifluoroacetamide (MSTFA) +1% trimethylchlorosilane (TMCS) (30 μL) were added to the samples, kept in room temperature for 1 h before heptane (30 μL, with 15 ng methylstearate /μL, injection standard) was added prior to GC-TOFMS analysis. A scheme for extraction and derivatization procedures is presented in figure S2.

**GC-TOFMS** **analysis**

1 μL aliquot was injected splitless by an CTC Combi Pal autosampler (CTC Analytics AG, Zwingen, Switzerland) into an Agilent 6890N gas chromatograph equipped with a 10 m × 0.18 inner diameter fused-silica capillary column chemically bonded with 0.18 μm DB 5-MS stationary phase (J&W Scientific, Folsom, CA). The injector temperature was set to 270 °C, the septum purge gas was turned on after 60 s at a flow rate of 20 mL min ^-1^. The initial column temperature was 70 °C and kept steady for 2 min. Temperature was increased by 40 °C min ^-1^ until a temperature of 320 °C was reached. Stop temperature was kept for 2 min before the oven was allowed to return and stabilize at the initial temperature. The gas flow rate thought the column was 1 mL min ^-1^. The column outlet was introduced into the ion source of a Pegasus III TOFMS (Leco® Corp., St Joseph, MI). The transfer line temperature was set to 250 °C and the ion source temperature was set to 200 °C. Ions were produced by a 70 eV electron beam (2.0 mA). Acceleration voltage was switched on after a 165 s solvent delay and masses were acquired between *m/z* 50-800, at a rate of 30 spectra s^-1^ for 445 s.

Runorder: To avoid confounding the metabolic variation related to ALS and ALS subtypes with runorder, the runorders were randomized prior to GC-TOFMS analysis in both sample sets (I and II). In set II the samples were randomized in pairs and the order within ALS and control was randomly selected to avoid bias. For evaluation of runorder bias against disease, see figure S3.

**Pre-treatment of GC-TOFMS data**

Hierarchical multivariate curve resolution (HMCR) was used to resolve chromatographic profiles and corresponding mass spectra from the acquired GC-TOFMS data. HMCR settings were optimized and datasets were selected for further multivariate analysis (filter length=3, noise limit=5, retention time precision=5, max peak shift=45). HMCR settings were optimized using a full factorial design in factors: filter length (0-5), noise limit (0-5) and retention time precision (3-7 scans). Max peak shift was kept constant at 45 scans. Optimal settings were rated by maximum number of resolved profiles, minimum number of bad spectra (calculated by in-house script), splitting of internal standard peaks (estimated by visual inspection) and library hit of endogenous metabolites (glutamine, valine, serine, tyrosine and pyroglutamic acid) and internal standards. For a scheme of the design see figure S4.

Missing values in the resolved data (i.e. zeroes meaning that the peak was not found in the sample) were checked against the injection standard methylstearate to estimate low concentration compounds as well as defining non-detected peaks in the samples. All resolved chromatographic profiles with more than 10% missing values were excluded from further analysis. Detected metabolites were identified by means of chromatographic retention indices and comparison of resolved mass spectra against in-house and public available mass spectra libraries (NIST 98 mass spectra library, the Umeå Plant Science Centre mass spectra library and the mass spectra library hosted by the Max Planck Institute in Golm (<http://csbdb.mpimp-golm.mpg.de/csbdb/gmd/gmd.html>)) or *de novo* identification. Chromatographic profiles tied to noisy mass spectra were checked and excluded if found to be inconsistent with peaks in the raw data. The areas under peaks remaining from the same metabolites were summed and unknown metabolites were matched against spectra from the same analysis and their areas were summed if discovered to be consistent with the same mass spectrum. Unknown metabolites were categorized (if possible) into chemical classes (i.e. fatty acid, amino acid etc.). Combined peaks (summed areas) were given the average retention index value of the original peaks.

Overlapping peaks (with internal standards) were computed for all subjects by an in-house MATLAB 7.3 (R2006b) based (Mathworks, Natick, MA) script developed for multiple AUC calculations of mass channels: isocitric acid *(m/z* 319), glutamic acid *(m/z* 248), cholesterol *(m/z* 329+368), hexadecanoic acid *(m/z* 313), proline *(m/z* 216), myristic acid *(m/z* 285), putrescine *(m/z* 200+214), salicylic acid *(m/z* 267), succinic acid *(m/z* 247), sucrose *(m/z* 451+437), glucose *(m/z* 319+229, non-specific mass channels), urea (3TMS) *(m/z* 261+276), urea *(m/z* 171+189+204).

The data were normalized by a procedure using the 11 internal standards eluting over the whole chromatographic time range and methylstearate (injection standard). The area under the chromatographic peak was calculated for all standards using unique mass channels. A principal component analysis using non-noisy mass channels (scaled to unit variance, non-centered) were calculated and the score value for each sample was used to normalize the resolved data by dividing each sample with the corresponding score value.

Prior to multivariate data analysis the internal standards, methylstearate and identified drugs (i.e. paracetamol and salicylic acid) were excluded and the processed metabolite data were mean centered and scaled to unit variance.

**Extraction of important metabolites from OPLS-DA modelling**

Important metabolites were highlighted in the pair-wise models by investigating the most influential metabolites in the model loadings (separating the ALS group from the control group) but also by identifying metabolites changing the most between the individual pairs (matched ALS-control) by listing the contribution for the variables on the model scores in standard deviations and sorting out metabolites showing the same direction in terms of disease related response in 2/3 or more of the sample pairs.

**Set III**

For set III the subjects in set I were predictedly resolved by HMCR using the same settings as for set II and the matched control data were subtracted from the corresponding ALS case data prior to multivariate analysis. Thus set III is built up by the same metabolites as originally were detected in set II. Three samples were found to be strong outliers (deviations were assessed to belong to methodological problems) in PCA and were removed before OPLS-DA modeling.
